# Effect of Damping on the Identification of Bridge Properties Using Vehicle Scanning Methods

**DOI:** 10.3390/s24175785

**Published:** 2024-09-05

**Authors:** Emrah Erduran, Semih Gonen

**Affiliations:** 1 Department of Built Environment, Oslo Metropolitan University, 0166 Oslo, Norway; 2 Department of Civil and Environmental Engineering, Universitat Politècnica de Catalunya, 08028 Barcelona, Spain; semih.gonen@upc.edu

**Keywords:** drive-by, system identification, bridges, structural health monitoring

## Abstract

Vehicle scanning methods are gaining popularity because of their ability to identify modal properties of several bridges with only one instrumentation setup, and several methods have been proposed in the last decade. In the numerical models used to develop and validate such methods, bridge damping is often overlooked, and its impact on the efficacy of vehicle scanning methods remains unknown. The present article addresses this knowledge gap by systematically investigating the effects of bridge damping on the efficacy of vehicle scanning methods in identifying the modal properties of bridges. For this, acceleration responses obtained from a numerical model of a bridge and vehicle are used. Four different scenarios are considered where vehicle damping, presence of road roughness, and traffic on the bridge are varied. Bridge damping is modeled using mass-proportional, stiffness-proportional, and Rayleigh damping models. The impacts of ignoring bridge damping or considering one of these damping models on the modal frequencies and mode shapes identified using the vehicle response are investigated by comparing the results. The outcomes of the numerical analysis show that ignoring bridge damping in vehicle scanning applications can significantly increase the efficacy of these methods. They also show that the identifiability of the bridge frequencies and bridge mode shapes from the vehicle response decreases significantly when bridge damping is considered. Further, the damping model used impacts which bridge modes can be identified because different damping models provide different modal damping ratios for each mode. The results highlight the importance of correctly simulating damping behavior of bridges, which is often ignored, to be able to correctly evaluate the efficacy of vehicle scanning methods, and they provide an important stepping stone for future studies in this field.

## 1. Introduction

To enhance bridge safety and facilitate intelligent maintenance practices, structural health monitoring (SHM) techniques have been under development for several decades [1]. Vibration-based methods are the most widely utilized among SHM techniques, largely because they are non-destructive and can assess the condition of the entire bridge. However, direct vibration-based SHM methods necessitate the installation of multiple accelerometers and data acquisition systems on each bridge being monitored [2,3]. More recently, efforts have been made to replace some of the accelerometers with computer vision techniques [4].

Despite significant advancements, the enormous number of bridges worldwide, numbering in the millions, makes it prohibitively expensive to install separate structural health monitoring (SHM) systems on each one. As an appealing alternative, vehicle-mounted sensors, commonly referred to as vehicle scanning methods (VSMs), offer a cost-effective solution by allowing the monitoring of multiple bridges using a single instrumentation setup with fewer sensors. The concept of vehicle scanning methods was first introduced by Yang et al. [5]. Most research on VSMs has concentrated on identifying the vibration frequencies of bridges by analyzing accelerations derived from numerical models [6,7,8,9,10,11,12]. Additional studies have sought to determine bridge mode shapes [13,14,15,16,17,18,19,20,21,22,23,24], identify and mitigate the negative impacts of surface roughness [25,26,27,28,29,30], and detect the presence of damage [31,32,33,34,35,36]. Although limited in number, laboratory and field experiments have been conducted to validate these theoretical and numerical findings [37,38,39,40,41,42,43]. Yang and Yang [44], and Yang et al. [45] provide comprehensive summaries of the most influential publications on vehicle scanning methods.

As can be seen from the presented literature, the majority of the VSM studies are based on numerical investigations. One common aspect of several numerical studies on vehicle scanning methods is the lack of damping in the numerical models of bridges. While damping in vehicles is generally considered in the numerical models, damping in the bridge is ignored, claiming its effect on the bridge and vehicle response compared with the forced vibration is insignificant [13,46]. Thus, numerical analysis that forms the basis of the data used to identify modal properties of bridges from accelerations recorded in vehicles are often conducted on undamped bridge models [5,6,10,13,20,25,29,35,46]. Recently, Erduran et al. [47] investigated the effect of damping models on masonry arch bridges and demonstrated the significance of this parameter. On the other hand, several studies in the field of earthquake engineering demonstrate the importance of the amount of damping and the damping models used in the numerical models on the response of structures under seismic loads [48,49,50,51,52,53,54]. It is also noted that the forced vibrations are in general much higher under seismic loads compared with their counterparts in vehicle–bridge interaction problems. Combining these two observations, i.e., the demonstrated effect of damping in various scenarios, we can pose a question on the validity of the assumption that bridge damping does not significantly affect the vehicle response and, in turn, the results of the vehicle scanning methods. Indeed, only a few studies have considered bridge damping in numerical analysis for vehicle scanning methods [17,21,33,55], but they neither investigated nor commented on the effects of damping on the results of VSM applications. Only a handful of recent articles have addressed the effects of damping on the mode shapes identified using vehicle scanning methods [22,23,56,57,58]. Of these, the method proposed in [56] assumes that the mode shapes identified are identical to the theoretical mode shapes, while the mode shape correction method proposed in [22] was later shown to work only in symmetrical structures [23]. Further, to the best of our knowledge, none of the studies has considered the effect of the damping model on identification of the modal parameters using VSMs. As such, the question we posed and the research gap remain open and unexplored.

To this end, this article systematically investigates the effect of bridge damping on the vehicle response obtained from numerical models used to simulate vehicle–bridge interactions. More specifically, we will focus on the effect of bridge damping on the efficacy of vehicle scanning methods in estimating the modal parameters of the bridges from the vehicle response. For this, we first examine the theoretical formulation of the vehicle–bridge interaction problem. Then, we conduct a numerical study that uses a numerical model of a 25 m long bridge and a single-degree-of-freedom system that simulates a vehicle crossing the bridge. The damping in the bridge and the damping ratio of the vehicle are then varied, and the impact of these two parameters on the vehicle response and on the identifiability of the bridge modal frequencies from the vehicle response is evaluated numerically. In addition to the modal frequencies, identification of the mode shapes using VSMs is highly desirable because mode shapes are often used in SHM applications such as finite element model updating and damage detection [59]. Thus, we investigate the impact of bridge damping and different damping models used to simulate bridge damping on the accuracy of a state-of-the-art vehicle scanning method in identifying the mode shapes of the bridge.

Research about the impacts of damping and damping models on structural behavior is limited. This limitation is more pronounced for structural health monitoring applications in general and vehicle scanning methods in particular. This article, for the first time, presents a systematic study that explores the impact of bridge damping in identifying modal properties of bridges using vehicle scanning methods. Further, it highlights the importance of correctly simulating damping behavior of bridges, which is often ignored, to be able to correctly evaluate the efficacy of vehicle scanning methods. Thus, it provides an important stepping stone for future studies in this field.

## 2. Analytical Evaluation

**Figure 1:**
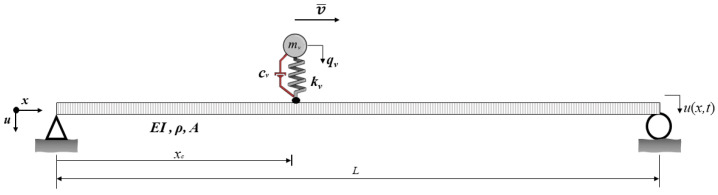
Mathematical model consisting of the bridge and the vehicle.

The equation of motion of a bridge during a vehicle crossing (Figure 1) can be written as:

(1) $$m{\ddot{u}}_{b}+c{\dot{u}}_{b}+ku_{b}=f_{c}\left(t\right)\delta \left(x-vt\right)$$

where m is the mass matrix, c is the damping matrix, k is the stiffness matrix, $u_{b}$ is the vector that denotes vertical displacements of the bridge, $f_{c}\left(t\right)$ is the contact force between the bridge and the vehicle, δ is the dirac delta function, and *v* is the velocity of the vehicle that is assumed to be constant throughout the analysis.

Similarly, the equation of motion of the single-degree-of-freedom system emulating the behavior of the vehicle is:

(2) $$m_{v}{\ddot{q}}_{v}\left(t\right)+c_{v}{\dot{q}}_{v}\left(t\right)+k_{v}\left(q_{v}-u_{c}\right)=0$$

where $m_{v}$, $c_{v}$, $k_{v}$ are the mass, damping coefficient, and stiffness of the single degree-of-freedom that simulates the vehicle, respectively. $q_{v}$ is the vertical displacement of the vehicle and $u_{c}$ is the vertical displacement at the contact point between the vehicle and the bridge.

The contact force and displacement between the bridge and vehicle can then be computed as:

(3) $$f_{c}\left(t\right)=-m_{v}g+k_{v}\left(q_{v}\left(t\right)-u_{c}\right)$$

(4) $$u_{c}=\left(u_{b}+r\right){|}_{x=vt}$$

where *g* is the gravitational acceleration and *r* is the road roughness. Combining Equations (2), (3), and (4), the equation of motion for the vehicle can be rewritten as:

(5) $$m_{v}{\ddot{q}}_{v}\left(t\right)+c_{v}{\dot{q}}_{v}\left(t\right)+k_{v}q_{v}=k_{v}\;\left(u_{b}+r\right){|}_{x=vt}$$

For a given vehicle and road profile, i.e., constant $m_{v}$, $c_{v}$, $k_{v}$, and *r*, it is clear from Equation (5) that the vertical response of the vehicle at any point in time depends solely on the bridge displacement at that moment, $u_{b}{|}_{x=vt}$.

Since the dynamic response of any system is impacted by the damping in the system, both in the forced vibration and free vibration state, the bridge damping matrix, c, should be expected to affect the bridge displacement response, $u_{b}$. Therefore, we can conclude that, keeping all other variables constant, the displacement response of the bridge, $u_{b}$, is expected to be impacted by the bridge damping matrix. As such, the displacements in a damped bridge, $u_{b}{|}_{x=vt}$, will be different compared with their counterparts in an undamped bridge, further affecting the vehicle response, as indicated by Equation (5).

Finally, the right side of the Equation (5) represents the force applied to the single degree-of-freedom system representing the vehicle, p(t). This forcing function has two components: bridge displacement and road roughness. For a given road roughness profile, the second component of the forcing function, p(t), remains constant when the bridge damping changes. On the other hand, the first component of p(t) that is based on the bridge displacement, as explained above, can be affected by the bridge damping. This, in turn, can lead to a decrease in the contribution of the bridge displacements on the vehicle response compared with the contribution of the road roughness. Considering that contamination of the vehicle response due to road roughness remains one of the biggest challenges that remain in vehicle scanning methods [44], and the increased contribution of the road roughness due to increased bridge damping can potentially aggravate this challenge even more. On the contrary, ignoring bridge damping can artificially increase the contribution of the bridge response to the vehicle response, leading to improved performance of the vehicle scanning methods, which may be potentially misleading depending on the road roughness profile and the level of damping in the bridge.

Although it is clear from the analytical formulation presented above that bridge damping is likely to impact the response of the vehicle, the level of the effect of damping on the vehicle response remains unknown. To quantify this impact, we conducted numerical analysis ignoring and considering bridge damping and using different damping models. The outcomes of this numerical study are summarized in the following sections.

## 3. Numerical Model

To assess the influence of bridge damping on vehicle dynamics, we developed a numerical model of a 25-meter-long, single-span bridge using Bernoulli beam theory. The bridge, simply supported at both ends, was discretized into 0.5-meter intervals. The material and cross-sectional characteristics of the bridge include a modulus of elasticity of 36GPa, a moment of inertia of $0.41\;m^{4}$, an area of $3.45\;m^{2}$, and a mass density of 8.625t/m. Through eigenvalue analysis, the bridge’s first three modal frequencies were identified as 3.5Hz, 12.2Hz, and 25.2Hz. The bridge was designed as a generic bridge that is representative of its kind. We ensured that the frequencies of the bridge were compatible with its geometry. The vehicle crossing the bridge was modeled as a single-degree-of-freedom system with a mass of 5 tons and a spring stiffness of $k_{v}=1500\;\mathrm{kN}/m$, resulting in a natural frequency of 2.65Hz.

**Figure 2:**
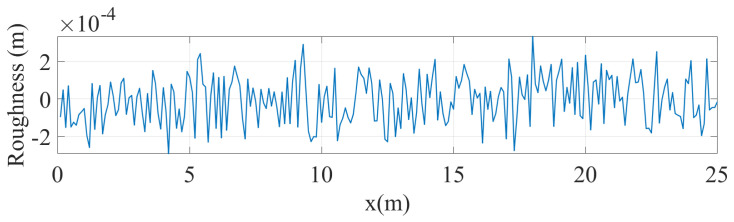
Road roughness profile.

Road roughness, which plays a crucial role in the acceleration response of a vehicle in motion, was also incorporated into the numerical model. A road roughness profile was generated using the power spectral density curve for Class A roughness, as defined by ISO 8608, which represents the smoothest category of road surfaces. The specifics of how the roughness profile was simulated are detailed in other sources, such as [21], and are omitted here for brevity. The roughness profile was specified at discrete intervals of 0.05 m (Figure 2), with linear interpolation used to estimate values when the vehicle was positioned between these points. To account for the finite contact length between the wheel and the road, a moving average filter was applied to the roughness profile during numerical analysis.

Four different vehicle damping coefficients, $c_{v}$, were used in the analysis to obtain vehicle damping ratios of $\xi_{v}=0\%,1.5\%,3\%,5\%$. While vehicle damping can simply be defined using a single coefficient, an approximate damping model needs to be used for the bridge, which is a multi degree-of-freedom system. One of the most widely used damping models in structural dynamic applications is the Rayleigh damping model and its two derivatives, mass-proportional damping and stiffness-proportional damping. Through Rayleigh damping, we can specify the modal damping ratios at two modal frequencies or, in case of mass- and stiffness-proportional damping, one modal frequency. The damping matrix, c, can be computed as:

(6) $$c=a_{0}m+a_{1}k.$$

The modal damping ratio of the $n^{th}$ mode with the natural frequency $w_{n}$ can then be computed as:

(7) $$\xi_{n}=\frac{a_{0}}{2}\frac{1}{w_{n}}\;+\;\frac{a_{1}}{2}w_{n}$$

The coefficients $a_{0}$ and $a_{1}$ can be determined from the specified modal damping ratios $\xi_{i}$ and $\xi_{j}$ for the $i^{th}$ and $j^{th}$ modes, respectively. If both modes are assumed to have the same damping ratio, ξ, then:

(8) $$a_{0}=\xi \frac{2w_{i}w_{j}}{w_{i}+w_{j}};\;\;\;a_{1}=\xi \frac{2}{w_{i}+w_{j}}$$

For mass- and stiffness-proportional damping, the modal damping ratio is specified only at one modal frequency, and the $a_{0}$ and $a_{1}$ coefficients are respectively computed as:

(9) $$a_{0}=2\xi_{i}w_{i}$$

(10) $$a_{1}=\frac{2\xi_{i}}{w_{i}}$$

**Figure 3:**
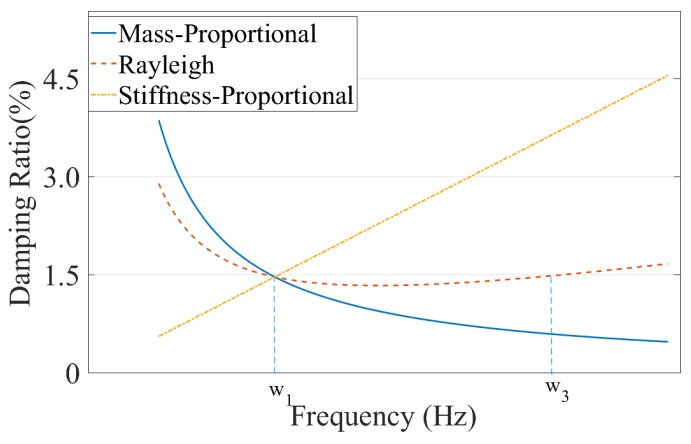
Variation of modal damping ratio with frequency.

The damping model and the corresponding modal damping ratio–natural frequency curve used in the numerical solution for the vehicle–bridge interaction problem can potentially impact the vehicle response. In particular, depending on the damping model used, some of the natural vibration modes of the bridge can be significantly damped out, reducing their contribution to the bridge response, and cannot be as prominent on the vehicle response as the others [60]. On the contrary, the damping model can favor some other modes by assigning lower modal damping ratios for those modes, increasing their contribution to the bridge response, and, as a result, to the vehicle response. Therefore, we decided to test all three damping models and evaluate their effect on the contribution of different bridge modes on the vehicle response. For the mass-proportional and stiffness-proportional damping models, we assigned a modal damping ratio of 1.5% to the first modal frequency. The Rayleigh damping model was anchored at the first and third natural frequencies with a modal damping ratio of 1.5% at these frequencies. The variation of the modal damping ratios with the natural frequencies obtained from the three damping models is presented in Figure 3.

## 4. Numerical Analysis

To investigate the effect of bridge damping on the vehicle response, we virtually drove the vehicle on the bridge with a constant speed of v=2 m/s. The Newmark method with parameters α=0.5 and β=0.25 and a time step of dt=0.001 s was used in the numerical solution. The contact between the vehicle and bridge was not lost during the analyses. Here, we should also note that the low vehicle speed was specifically chosen because it leads to better modal identification results, as shown by numerous examples in the literature summarized in the Introduction section. Thus, we chose to focus on and highlight the effects of damping in the case where VSM methods provide the best modal parameter estimates.

We considered four separate case studies to investigate the effects of the most important parameters, namely bridge damping, road roughness profile, and vehicle damping. In the first case, we ignored both the damping in the vehicle and the road roughness. In the second case, we continued to ignore vehicle damping but added road roughness. For the third case, we varied the vehicle damping to evaluate its effect on the identifiability of bridge frequencies from the response of a vehicle on a rough road, while in the last case we added the effect of existing traffic by sending a lead vehicle to pre-excite the bridge before the instrumented vehicle entered onto the bridge. In all of the four cases, we considered each of the damping models used for the bridge and also ignored the bridge damping for comparison.

### 4.1. Case I: Undamped Vehicle without Road Roughness

**Figure 4:**
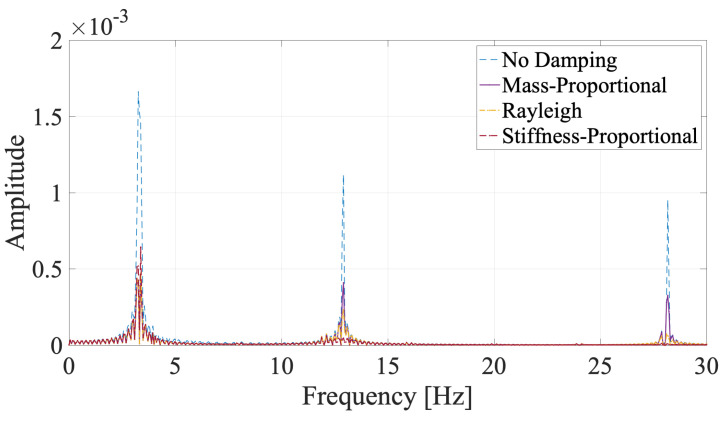
Fourier amplitude spectrum of the vibrations at the quarter-span of the bridge for different damping models.

We first look at the effect of bridge damping and the damping models on the behavior of the bridge. For this, the Fourier amplitude spectrum (FAS) of the vibrations created by the instrumented vehicle at the quarter-span of the bridge was computed and plotted in Figure 4. We chose to present the results at the quarter-span point to be able to avoid the nodal points in the first three vibration modes. The FAS of the vibrations at the quarter-span point clearly shows that ignoring bridge damping leads to significantly higher energy at each of the modal frequencies. Further, the three damping models used in the analysis lead to almost identical energy levels in the first mode because the modal damping ratio at this mode is set to $\xi_{1}=1.5\%$ for all three damping models. However, the energies in the vibrations at the second and, especially, the third modal frequencies differ significantly from each other for different damping models. Due to the very high damping ratio assigned by the stiffness-proportional damping model, the third mode has virtually no contribution to the vibrations on the bridge, while the contribution of the second mode is also limited for this damping model. On the contrary, the contribution of the third mode relative to the first mode is higher for the mass-proportional damping because the modal damping ratio decreases with an increase in the modal frequency for the mass-proportional damping model; see Figure 3. The Rayleigh damping model produces results that are between the mass-proportional and stiffness-proportional damping. Considering that vehicle scanning methods rely on the bridge vibrations to identify the bridge modal properties, the FAS of the bridge vibrations suggests that identification of the second and third modes using VSMs can be easier if mass-proportional damping is used in the analysis instead of the other two damping models. More importantly, the significantly higher energy in all three modes achieved by ignoring the bridge damping can, potentially, lead to easier identification of each of the modes using VSMs.

**Figure 5:**
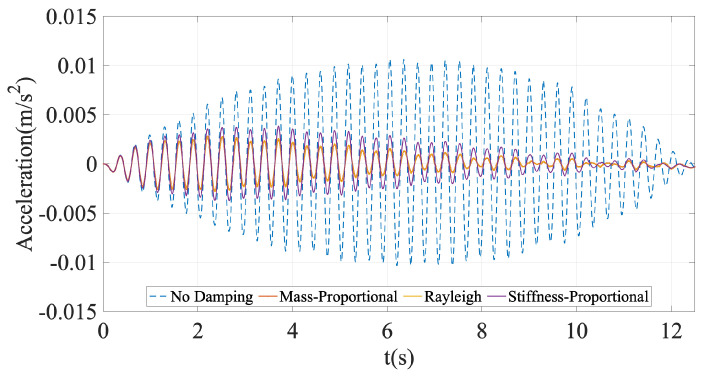
Acceleration response of the vehicle for different damping models.

Figure 5 shows the accelerations recorded on the vehicle using different bridge damping models. When the damping in the bridge is ignored, the acceleration response of the vehicle is much higher, particularly towards the middle of the motion. Immediately after the vibrations on the bridge commence, the vehicle responses for all four different cases are very similar to each other. Approximately two seconds into the motion, the accelerations recorded on the vehicle on the damped bridge starts to deviate significantly from those recorded on the undamped bridge, highlighting the impact of bridge damping on the vehicle response. When we focus only on the three different damping models and their effects on the vehicle response, we see that they are very close to each other with Rayleigh damping leading to slightly higher vehicle response.

Next, we evaluated the effect of bridge damping on the contact point response. The contact point response has previously been documented to significantly outperform the vehicle response in identifying the frequencies and mode shapes of bridges using vehicle scanning methods [5,20,21,24]. The contact point response, which cannot be measured in a field test, can be computed by differentiating Equation (2):

(11) $${\ddot{u}}_{c}=\frac{1}{k_{v}}\;\left(m_{v}\;\frac{d^{2}{\ddot{u}}_{v}}{dt}+c_{v}\;\frac{d^{2}{\dot{u}}_{v}}{dt}+k_{v}\;{\ddot{u}}_{v}\right)$$

The first and second derivatives of the vehicle accelerations can be computed using the central difference method, considering that the vehicle accelerations are recorded at discrete data points.

**Figure 6:**
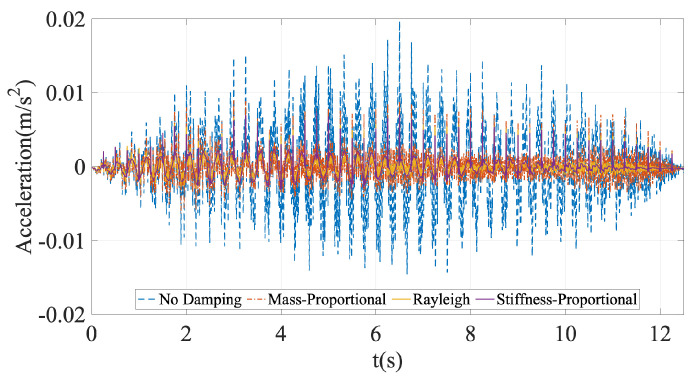
Contact point accelerations for different damping models for the undamped vehicle.

**Figure 7:**
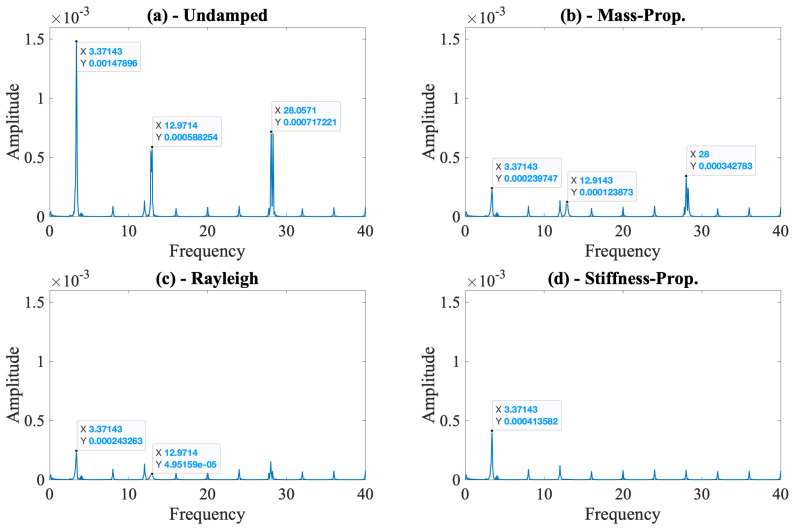
Fourier amplitude spectrum of the contact points for different damping models for the undamped vehicle.

Similar to the vehicle response, ignoring the damping in the bridge leads to much higher accelerations at the contact point; see Figure 6. Computing the Fourier amplitude spectrum of the contact point response sheds more light on the effect of bridge damping on the CP response. The FAS plotted in Figure 7 shows that the frequency content of the contact point response for different damping models follows the same trend as its counterpart for the bridge response depicted in Figure 4. We chose to plot the FAS separately for each damping model to be able to judge the visibility of the modal frequencies of the bridge on the Fourier spectrum of the contact response for each damping model. At all three bridge frequencies, ignoring bridge damping leads to a much higher energy in the contact point response. Following the discussion in Section 2, the decreased energy at each modal frequency due to bridge damping can potentially be crucial when the effect of road roughness is included in the upcoming cases.

When we focus only on the three damped cases, we can clearly see the effect of the damping model used in numerical analysis on the frequency content of the contact point response. While the energy at the first modal frequency remains unaffected by the damping model, the energy at the higher modes depends significantly on the damping model used in the numerical analysis. Mass-proportional damping provides the lowest damping at the third modal frequency and, thus, the contact point response has a much higher energy at the third modal frequency when this damping model is used. On the other hand, stiffness-proportional damping, which provides much higher damping at the third modal frequency, leads to the lowest energy at this frequency in the Fourier spectrum compared with the other damping models. As such, the efficacy of the vehicle scanning methods that use the contact point response in detecting the properties of the higher modes of the bridge can significantly be impacted by the damping model used in the numerical analysis.

In Figure 7, we can also observe energy at the multiples of 4 Hz. As indicated above, the bridge is discretized at 0.5 m intervals. When the vehicle travels with a speed of 2 m/s, it crosses a node every 0.25 s, i.e., at a frequency of 4 Hz. As such, these frequencies correspond to the vehicle crossing the nodes of the bridge and are numerical artifacts. These frequencies can be removed either by filtering the signal or by using a much denser mesh so that the frequency of crossing a node increases and moves out of the range of interest.

### 4.2. Case II: Undamped Vehicle—Road Roughness Class A

In the next case, we continue ignoring the damping in the vehicle but we add the effect of the road roughness by applying the roughness profile provided in Figure 2.

**Figure 8:**
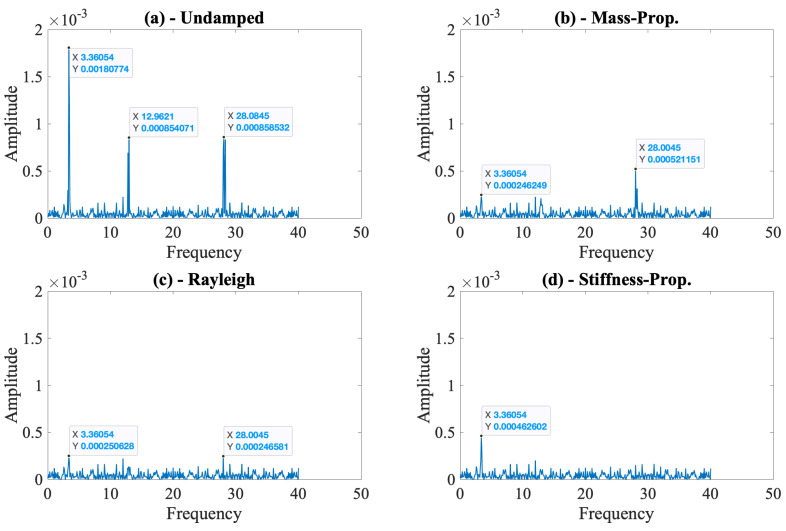
Fourier amplitude spectrum of the contact point response of the undamped vehicle for different bridge damping models considering road roughness class A.

Considering that the contact point response was previously shown to provide the best results for identification of modal properties of bridges using vehicle scanning methods [20,44], we focus only on this parameter for the rest of the article. We computed the Fourier amplitude spectrum of the contact point response for each bridge damping model, along with the undamped bridge, and plotted the spectra separately in Figure 8. The scale of each subplot in Figure 8 is the same, allowing the comparison of the spectral amplitudes at each modal frequency computed for the different damping models.

In Figure 8, we can clearly observe the effect of ignoring the bridge damping on the FAS of the contact point response. Comparing Figure 8a with Figure 8b–d shows that the higher energy at each modal frequency for the undamped bridge leads to a clear identification of each of the three modal frequencies for this case, even in the presence of road roughness. On the other hand, the combined effects of road roughness and bridge damping make it more difficult to identify the modal frequencies of the bridge for all three damping models used; see Figure 8b–d. The most prominent peaks in the FAS for the damped bridge are at the third mode frequency for the mass-proportional damping model (Figure 8b) and at the first mode frequency for the stiffness-proportional damping model (Figure 8d). Further, while it is still possible to recognize all the three modal frequencies for the mass-proportional and Rayleigh damping models, the third mode frequency and, to some extent, the second mode frequency are virtually impossible to identify when the stiffness-proportional damping model is used. In summary, Figure 8 shows that ignoring bridge damping in the numerical models used to develop or assess vehicle scanning methods is likely to lead to falsely improved efficacy in identifying the modal frequencies of the bridge because of the artificially increased amplitude at each of the modal frequencies. Further, when bridge damping is considered, the damping model used in the numerical model is also likely to affect which of the modes can be identified from the FAS of the CP response.

### 4.3. Case III: Damped Vehicle—Road Roughness

To have the most realistic numerical model, we need to consider three parameters together: bridge damping, road roughness, and vehicle damping. In this section, we add the last of these parameters by modeling the vehicle damping. In addition to the undamped vehicle, we consider three vehicle damping levels: 1.5%, 3%, and 5%. Numerical analysis is repeated for each combination of bridge damping model and vehicle damping, and the contact point responses are acquired. Considering we have now four different damping models for the bridge and four separate damping values for the vehicle, it is not practical to evaluate the FAS of each case separately, as we have 16 total cases. Therefore, we define a new parameter to quantify the presence of each modal frequency in the FAS of the contact response. This parameter is defined as the ratio of the area under the Fourier spectrum at the given modal frequency to the area of the entire spectrum. We will use the term *relative energy* at the given frequency to refer to this term, which represents the ratio of the energy at that modal frequency to the total energy in the contact point response. The higher the relative energy at a particular frequency is, the more prominent is the associated frequency component in the Fourier spectrum.

**Figure 9:**
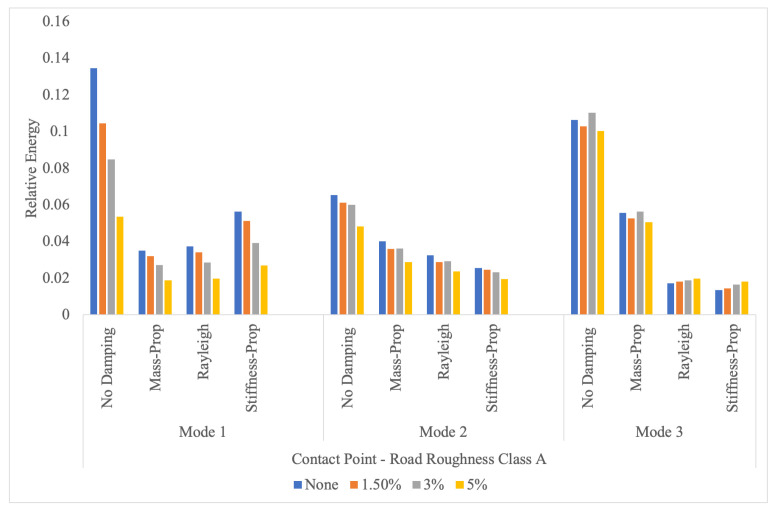
Relative energy at the first three modal frequencies in the contact point response for different vehicle damping values and bridge damping models considering road roughness class A.

Figure 9 shows the relative energy values at the first three bridge frequencies of the contact point response for different vehicle damping values and bridge damping models. For each mode, the horizontal axis shows the bridge damping model, while the different vehicle damping values are represented by different colored bars. The effect of the vehicle damping is highest for the first mode and when the damping of the bridge is neglected. The effect of vehicle damping is highest for the undamped bridge model because, in this case, the only damping in the system is provided by the vehicle damping amplifying its impact on the contact point response. It is also interesting to observe that the impact of vehicle damping on the relative energy at the modal frequencies decreases with an increase in the mode number. If we focus on the third mode, we can observe that the vehicle damping has no significant impact. This is very different from the first mode where the vehicle damping has a considerable impact on the relative energy. This can be explained by the frequencies of the vehicle and the bridge. The vehicle frequency is 2.65 Hz, which is relatively close to the first mode frequency of the bridge (3.5 Hz) while the third mode frequency (20.2 Hz) is very far away from the vehicle frequency. Thus, adding damping to the vehicle, which has a natural frequency of 2.65 Hz, is likely to damp out some of the energy transferred from the bridge to the vehicle at the frequencies close to the vehicle frequency, such as the first modal frequency. On the other hand, the energy at the third mode frequency is not impacted by the vehicle damping because its vibration frequency is very different from the vehicle frequency.

### 4.4. Case IV: Damped Vehicle—With Road Roughness and Traffic

In the final case, we considered the effects of existing traffic by sending a lead vehicle before the instrumented vehicle enters the bridge. The objective of this case is to observe whether the existing traffic can alleviate the negative effects of the road roughness on modal frequency identification. The lead vehicle has a mass of 20 t, and we adjusted its stiffness and damping coefficients such that both vehicles have the same frequency, 2.65 Hz, and damping ratio, 1.5%. The lead vehicle and the instrumented vehicle travel with the same speed, v=2 m/s. The instrumented vehicle is sent 12.5 s after the lead vehicle, i.e., it starts its trip as the lead vehicle completes its trip on the bridge.

**Figure 10:**
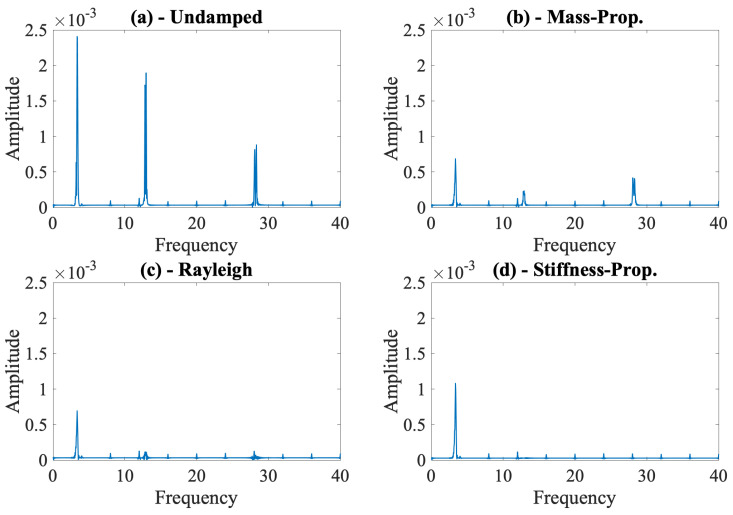
FAS of the contact point response for different bridge damping models considering road roughness class A and traffic.

The instrumented vehicle enters a bridge that is already oscillating thanks to the dynamic loading provided by the lead vehicle. Further, these initial oscillations are virtually unaffected by the road roughness. Comparing the cases with and without traffic, Figure 8 and Figure 10, it is clear that setting the bridge in motion before the instrumented vehicle starts its trip is an efficient way to decrease the adverse effects of road roughness because the existing oscillations amplify the energy at the modal frequencies of the bridge compared with the energy at the frequencies related to the road roughness. However, Figure 10 also shows that the impact of the lead vehicle is much higher when bridge damping is ignored because the oscillations created by the lead vehicle and picked up by the instrumented vehicle are not damped out by the bridge damping. While the first modal frequency can be identified clearly for all damping models, the second and third modal frequencies are very difficult to identify when Rayleigh or stiffness-proportional damping models are used, showcasing the importance of bridge damping and the damping model used on the efficacy of the VSM methods. In summary, the lead vehicle alleviates the effect of road roughness in the frequency content of the contact point response, but the effect of bridge damping remains prominent, indicating that ignoring bridge damping in numerical models can lead to artificially increased efficacy for frequency identification using VSMs.

Identifying only vibration frequencies of bridges is rarely enough to evaluate their condition. Mode shapes are often used in structural health monitoring applications such as finite element model updating and damage detection. Thus, identifying bridge mode shapes using VSMs is of great interest and, in the next section, we will investigate how bridge damping impacts mode shape identification using vehicle scanning methods.

## 5. Mode Shape Identification

We evaluate the effect of bridge damping on the mode shapes identified from the contact point response of the vehicle by considering three scenarios where the bridge has (i) a smooth profile, (ii) a class A roughness profile, and (iii) a rough profile with induced traffic. For this, we focused on the case where the vehicle damping is set to 1.5% of its critical damping. Once we obtained the CP response and identified the modal frequencies from the FAS of the CP response, as discussed in the previous sections, we decomposed the CP response to its modal components using signal processing. In this article, we used the variable mode decomposition method adopted by [20,23] that allows obtaining the modal components of a vibration signal by calculating all the modal waveforms and their central frequencies. After computing the modal components of the signal, the mode shapes are constructed using the instantaneous amplitudes obtained from the Hilbert Transform (HT). This method mirrors other studies used for mode shape identification from the contact point response [20,21]. The reader is referred to these articles for further details on mode shape identification from the CP response. There are other methods used in mode shape identification in addition to the method applied here. For those who are interested in the differences of these methods, we evaluated the efficacy of these methods in identifying the mode shapes of bridges in a recent article [24].

**Figure 11:**
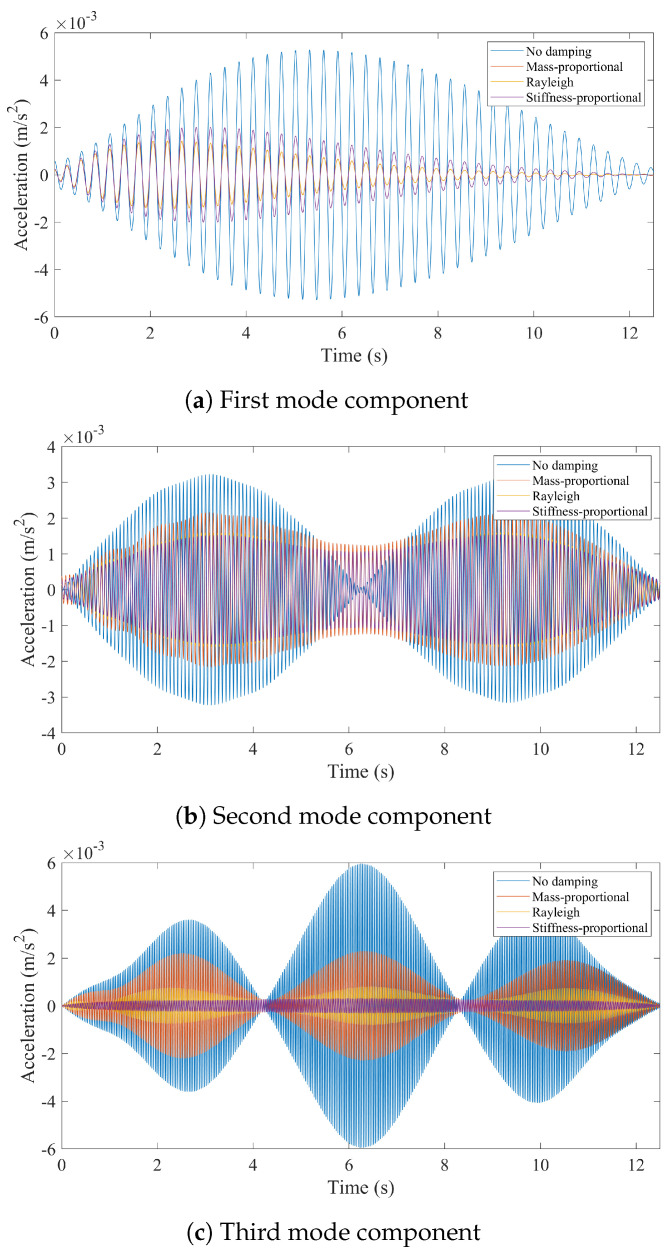
First three modal components identified from the contact point response for smooth road profile.

**Figure 12:**
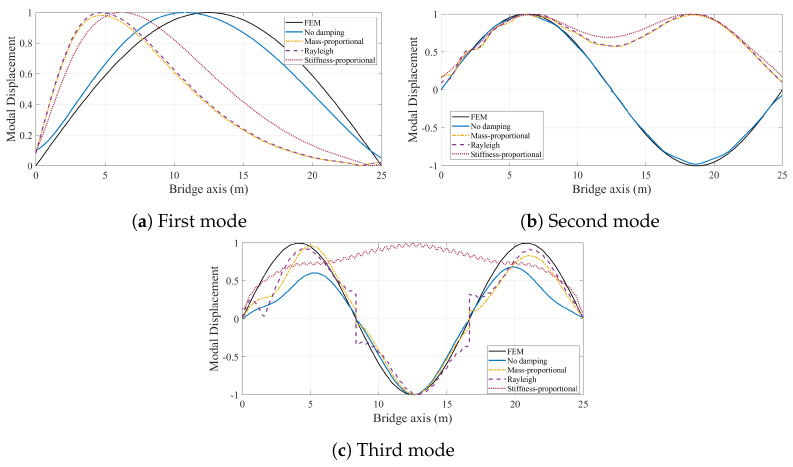
Comparison of first three mode shapes identified from the contact point response with the analytical solution for smooth road profile.

First, we consider the case where the bridge is assumed to have a smooth road profile. Figure 11 depicts the modal components for the first three modes of the bridge obtained from the CP response of the vehicle for different bridge damping models. The effect of bridge damping in the modal components of the CP response is significant for each of the three modes. When bridge damping is ignored, the modal components of the first two modes resemble the analytical mode shapes of the bridge, while those obtained with bridge damping considered are unsymmetrical. Indeed, when the mode shapes are extracted using HT, which are plotted in Figure 12, it is clear that the first two mode shapes can be identified with excellent accuracy when bridge damping is ignored.

In Figure 12, all mode shapes are normalized so that the maximum modal displacement is unity. This figure clearly depicts that, even in the absence of road roughness, bridge damping significantly distorts the mode shapes obtained from the contact point response. For the first mode, the three damping models provide very similar mode shape estimates because the damping ratio at the first modal frequency is constant for all three models, but all three damping models provide mode shapes estimates that are unsymmetrical and far from the analytical mode shape, which is also plotted in Figure 12a.

The modal components for the second vibration mode obtained from the CP response are plotted in Figure 11b. We should note that we amplified the modal components for the damped cases five times to be able to make their details visible compared with those obtained from the undamped bridge. For the second mode, all three damping models provide symmetrical mode shape estimates as depicted in Figure 12b. However, they cannot capture the nodal point, i.e. the point where the modal displacement is zero. Recalling that the second modal component extracted from the three damping models was multiplied by five in Figure 11b, very low amplitudes of the second modal component obtained when damping is considered are likely the reason for the poor mode shape estimates obtained in these cases. On the contrary, the amplitudes of the modal component extracted from the CP are much higher when bridge damping is ignored, and the second mode shape can be estimated with great accuracy for the undamped bridge. A similar observation can also be made for the third mode shape estimation when stiffness-proportional damping model is used. The modal components for different damping models shown in Figure 11c show that the amplitudes of the third modal component for the stiffness-proportional damping model are much lower compared with both the no damping case and mass-proportional damping model because the stiffness-proportional damping model provides a much higher modal damping ratio for the third mode than the mass-proportional damping model; see Figure 3. As such, the stiffness-proportional damping model cannot capture the nodal points of the third mode while the other damping models can; see Figure 12c. As such, the third mode shape estimate from the stiffness-proportional damping model is much poorer compared with the estimates obtained using the other two damping models.

**Figure 13:**
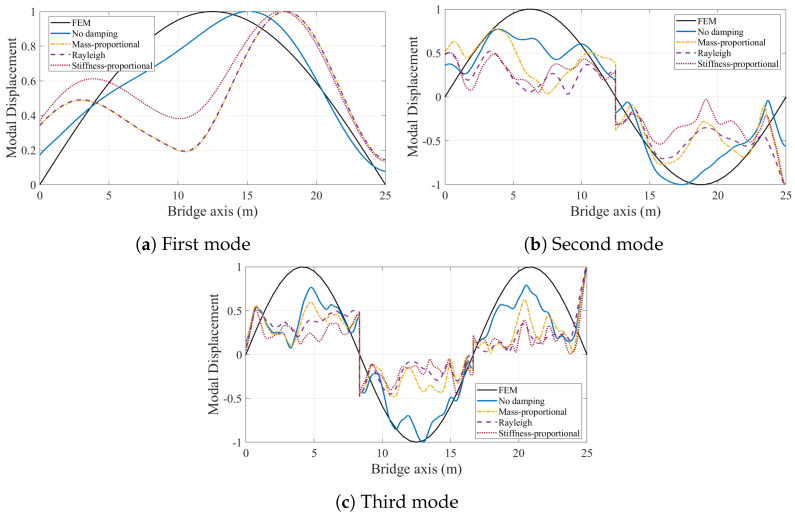
Mode shapes identified from the contact point response of the vehicle travelling on rough road.

Next, we repeated the modal identification process for the case where road roughness is considered. Figure 13 presents the normalized mode shapes identified from the contact point response for different bridge damping models. Also plotted in the figure are the mode shapes computed from the finite element model. For all cases, the impact of the road roughness on the accuracy of the mode shapes identified using VSMs is clear. However, ignoring the bridge damping still provides mode shapes that are relatively close to the theoretical mode shapes. On the other hand, including bridge damping in the numerical model leads to much poorer estimates for all three damping models considered.

**Figure 14:**
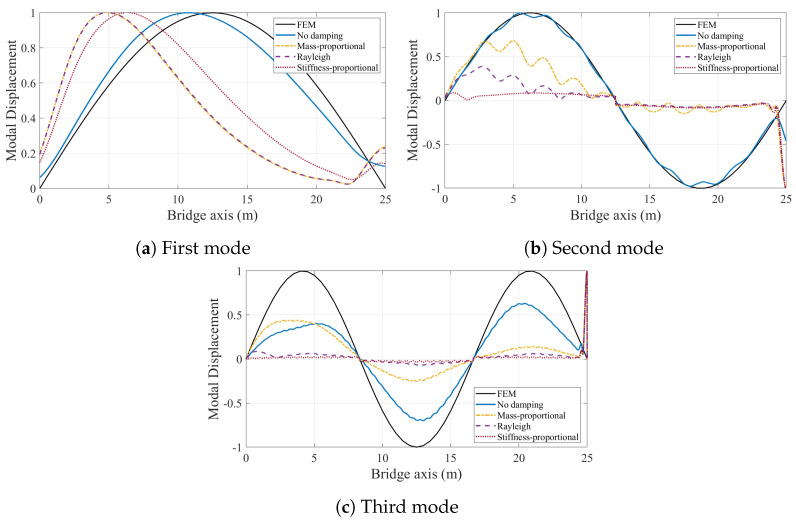
Mode shapes identified from the contact point response of the vehicle travelling on rough road considering vibrations from existing traffic.

When we add a lead vehicle to alleviate the adverse effects of road roughness, the mode shape estimates from the VSMs are significantly improved; see Figure 14. This improvement is much more pronounced for the undamped case, where pre-exciting the bridge via a lead vehicle before the instrumented vehicle enters the bridge eliminates virtually all adverse effects of road roughness; see Figure 12 and Figure 14. Figure 12 and Figure 14 further show that, for the other three damping models, the improvement in the first mode shape is also very significant and the first mode shape estimates from the two cases where the road roughness is ignored and where the road roughness is considered but the bridge is excited via a lead vehicle are nearly identical.

Sending a lead vehicle clearly improves the mode shape estimates for the second and third mode shapes compared with the case where road roughness is considered but no lead vehicle is included (see Figure 13 and Figure 14) for the three cases where the damping model is considered. This improvement is particularly high for the mass-proportional damping and reduces significantly when the stiffness-proportional component is added, either on its own or as part of the Rayleigh damping. However, the improvement in the first mode estimate provided by the lead vehicle is considerably higher compared with the improvement in the second and third mode estimates. This can be explained by the fact that the bridge used in this study is a single-span bridge and its behavior is dominated by its first mode. Hence, the oscillations provided by the lead vehicle are likely to have a much higher contribution from the first mode compared with the other modes, making the identification of the first mode from the CP response of the instrumented vehicle much easier.

The results of the numerical study to identify the modal mode shapes of a simply supported bridge using VSMs summarized above show that ignoring bridge damping is highly likely to artificially improve the efficacy of the VSMs in estimating the mode shapes of the bridge. We use the term artificial to describe the improvement in the efficacy of the VSMs due to ignored bridge damping because, physically, bridge damping is always present. Thus, considering bridge damping in the numerical models used to develop VSMs for mode shape estimation is crucial to be able to correctly evaluate the efficacy of the developed method.

## 6. Concluding Remarks

In this study, we examined the effects of bridge damping on modal identification using vehicle scanning methods. The study was motivated by the fact that several studies that focus on VSMs for modal identification ignore bridge damping because it is assumed to have no significance on the forced vibration response, even though damping is inherently present in every structure. We investigated the validity of this assumption through a numerical study, where we used the contact point response of the vehicle crossing undamped and damped bridge models to identify the modal properties of the bridge. Further, we used different damping models to evaluate if the damping model used has any effect on the modal identification using VSMs. We can summarize the main conclusions drawn from the results of this numerical study as:

- The frequency content of the contact point response is significantly affected by the bridge damping and the damping model used to simulate it even when road roughness is ignored. While all three modal frequencies are clearly visible in the FAS of the bridge when bridge damping is ignored, their amplitudes becomes significantly lower when bridge damping is considered.
- The impact of bridge damping on frequency content of the contact point response becomes even more clear when road roughness is included in the numerical analysis.
- Different damping models, assuming that they are anchored at the same modal damping at the first modal frequency, provide different damping levels at the higher modes. As such, the visibility of the modal frequencies in the FAS of the contact point response depends on the bridge damping model. While mass-proportional damping favors higher modes because of reduced damping at higher frequencies, the stiffness-proportional damping model and to some extent Rayleigh damping model suppress the higher mode frequencies.
- Pre-exciting the bridge before the instrumented vehicle starts its trip by sending a lead vehicle significantly improves the visibility of the modal frequencies on the FAS of the contact point response.
- The effect of bridge damping is even more pronounced in mode shape identification using VSMs. For the smooth road profile, considering bridge damping in the numerical model leads to an unsymmetrical first mode shape. As such, the accuracy of the identified first mode can unrealistically be improved if bridge damping is not considered in the numerical model. For the second mode, the identified shapes from the contact point response are not accurate enough when bridge damping is considered because the nodal point in the second mode cannot be captured from the contact point response when bridge damping is considered. While the mass-proportional and Rayleigh damping models provide relatively accurate estimates for the third mode shape when the road is assumed to be smooth, the high modal damping for the third mode provided by the stiffness-proportional model leads to very poor estimates for the third mode shape.
- Once road roughness is added, the mode shapes estimated by the VSMs considering bridge damping become very poor, especially for the second and third modes. Although the mode shape estimates acquired by ignoring the bridge damping also suffer from road roughness to some extent, the impact is much more significant for models that consider bridge damping.
- Crossing of a lead vehicle prior to the instrumented vehicle significantly improves the mode shape estimates.
- Compared with bridge damping, vehicle damping has a relatively lower impact of the frequency content of the contact point response.

This article shows that ignoring bridge damping in numerical analysis where the aim is identifying the modal characteristics of the bridge using VSMs is very likely to lead to artificially improved results. Since damping is present in every bridge, this phenomenon needs to be properly addressed in the numerical studies to ensure that the efficacy of the VSMs can be evaluated correctly. In this article, we evaluated three of the available damping models because they are arguably the most commonly used models in numerical modeling of structures. In future studies, the effects of different damping approaches such as non-viscous damping should be studied.

This study, similar to the majority of the studies so far on vehicle scanning methods, is a numerical study. While numerical studies are useful to understand the effects of several parameters on the proposed methods, it cannot, despite our best efforts, emulate all the practical limitations of real-world applications, which have recently been excellently summarized in [61]. Therefore, laboratory and field tests are crucial to further understand the effect of bridge damping on vehicle scanning methods.

Finally, we would like to also point out that there is a significant knowledge gap in the damping behavior of bridges under traffic loads. The majority of the field tests that aim to identify bridge damping levels are limited to ambient vibration tests. However, the damping levels obtained from ambient vibrations are not necessarily representative of the damping levels under forced vibrations because of the difference in the amplitude of these two vibration types [47]. Thus, it is difficult to judge which of the damping models evaluated in this article best represents the damping behavior of bridges for vehicle scanning applications. More field tests under correct vibration amplitudes are necessary to understand the damping level of bridges under forced vibrations created by vehicles.

## Data Availability

Data used in the study is available upon request.
